# Chlorogenic Acid Prevents Osteoporosis by Shp2/PI3K/Akt Pathway in Ovariectomized Rats

**DOI:** 10.1371/journal.pone.0166751

**Published:** 2016-12-29

**Authors:** Rong Ping Zhou, Si Jian Lin, Wen Bing Wan, Hui Ling Zuo, Fen Fen Yao, Hui Bing Ruan, Jin Xu, Wei Song, Yi Cheng Zhou, Shi Yao Wen, Jiang Hua Dai, Mei Lan Zhu, Jun Luo

**Affiliations:** 1 Orthopaedic Department, The Second Affiliated Hospital of NanChang University, NanChang, JiangXi, China; 2 Regeneration and Rehabilitation Engineering Research Institute on Bone and Nerve of JiangXi, NanChang, JiangXi, China; 3 Orthopaedics Research Institute of Jiangxi, NanChuang University, NanChang, JiangXi, China; 4 Rehabilitation Department, The Second Affiliated Hospital of NanChang University, NanChang, JiangXi, China; Harvard Medical School/BIDMC, UNITED STATES

## Abstract

*Cortex Eucommiae* is used worldwide in traditional medicine, various constituents of *Cortex Eucommiae*, such as chlorogenic acid (CGA), has been reported to exert anti-osteoporosis activity in China, but the mechanism about their contribution to the overall activity is limited. The aims of this study were to determine whether chlorogenic acid can prevent estrogen deficiency-induced osteoporosis and to analyze the mechanism of CGA bioactivity. The effect of CGA on estrogen deficiency-induced osteoporosis was performed *in vivo*. Sixty female Sprague-Dawley rats were divided randomly among a sham-operated group and five ovariectomy (OVX) plus treatment subgroups: saline vehicle, 17α-ethinylestradiol (E2), or CGA at 9, 27, or 45 mg/kg/d. The rats’ femoral metaphyses were evaluated by micro-computed tomography (μCT). The mechanism of CGA bioactivity was investigated *in vitro*. Bone mesenchymal stem cells (BMSCs) were treated with CGA, with or without phosphoinositide 3-kinase (PI3K) inhibitor LY294002. BMSCs proliferation and osteoblast differentiation were assessed with 3-(4,5-dimethyl-2-thiazolyl)-2,5-diphenyl-2-H-tetrazolium bromide (MTT) and alkaline phosphatase, with or without Shp2 interfering RNA (RNAi). The results display that CGA at 27 and 45 mg/kg/day inhibited the decrease of bone mineral density (BMD) that induced by OVX in femur (*p*< 0.01), significantly promoted the levels of bone turnover markers, and prevented bone volume fraction (BV/TV), connectivity density (CoonD), trabecular number (Tb.N), trabecular thickness (Tb.Th) (all *p*< 0.01) to decrease and prevented the trabecular separation (Tb.Sp), structure model index (SMI)(both *p*< 0.01) to increase. CGA at 1 or 10 μM enhanced BMSC proliferation in a dose-dependent manner. CGA at 0.1 to 10 μM increased phosphorylated Akt (p-Akt) and cyclin D1. These effects were reversed by LY294002. CGA at 1 or 10 μM increased BMSC differentiation to osteoblasts (*p*< 0.01), Shp2 RNAi suppressed CGA-induced osteoblast differentiation by decreasing Shp2, p-Akt, and cyclin D1. This study found that CGA improved the BMD and trabecular micro-architecture for the OVX-induced osteoporosis. Therefore, CGA might be an effective alternative treatment for postmenopausal osteoporosis. CGA promoted proliferation of osteoblast precursors and osteoblastic differentiation of BMSCs via the Shp2/PI3K/Akt/cyclin D1 pathway.

## Introduction

Osteoporosis is an osteometabolic disease characterized by substantial loss of bone mass and micro-architectural deterioration of bone tissue, which reduces bone quality and strength leading to an increase in bone fracture risk. In healthy bone, remodeling activity retains bone quality and produces bone that can adapt to mechanical forces. The osteoclastic resorption phase of bone remodeling is short, and the osteoblastic bone formation phase is long; thus, any increase in the bone remodeling rate will result in a loss of bone mass[1]. Menopause results in accelerated bone remodeling along with an uncoupling of bone resorption and formation. That is, more bone is reabsorbed by osteoclasts than is replaced by osteoblasts on the trabecular bone surface, resulting in net bone loss[2, 3].

Postmenopausal osteoporosis is an increasing global health concern that currently affects about 200 million people worldwide. In 2014, the prevalence of Osteoporosis reached almost 14 million people in the USA alone[4]. According to a study of patients at multiple centers in China, the incidence of osteoporosis was 15.5% among 50- to 59-year-old patients and 81% among 80- to 89-year-old patients, with total incidence rates of 28% in the vertebral column, 15% in the femur, and 31% in the vertebral column and femur among 50- to 89-year-old patients[5]. Osteoporosis incidence increases markedly with increasing age, especially in women. Osteoporosis-related fractures are common among postmenopausal and elderly women, who experience age-related losses in bone mineral density (BMD), a phenomenon that has been linked to increased risk of fractures and disability[6].

Low estrogen is the most common causative factor of osteoporosis in postmenopausal women. As we know that estrogen clearly reduces bone resorption [7], and these effects are mediated by an inhibition of osteoclast development and activity as well as an increase in osteoclast apoptosis [8]. Estrogen deficiency is associated with an increase in bone remodeling [9], and the associated increase in bone resorption is accompanied by a coupled increase in bone formation at the tissue level [8]. It is reported that hormone replacement therapy (HRT) is used for osteoporosis prevention, while antiresorptive or anabolic drugs are used for treatment. Pharmacological agents reduce the risk of osteoporotic fractures by increasing BMD. Traditional anabolic drugs (e.g., growth hormone, sodium fluoride, anabolic steroids, etc.) stimulate bone growth, and antiresorptive drugs (e.g., calcitonin, bisphosphonates, etc.) may prevent further bone loss. However, the cost and side effects of many of these drugs can be burdensome. For example, studies from the Women's Health Initiative revealed that long-term HRT increases the risk of malignancy in reproductive organs, such as breast cancer, and orther risk factors for cardiovascular disease and VTE[10]. The most common side effects of oral bisphosphonates are mild upper gastrointestinal symptoms, the usual i.v. bisphosphonate of choice may occur hypocalcaemia and an acute-phase reaction (fever, myalgia, lymphopenia, elevated CRP) due to pro-inflammatory cytokine (TNFa, interferon-g and IL-6) release by an activated sub-set of T-cells, long-term used bisphosphonate may induce suppression of bone turnover, which impairs the natural mechanism of bone repair, concerns have been raised with regard to potentially increased risk of osteonecrosis of the jaw (ONJ) and atypical fractures. In view of the considerations to these disadvantages that have motivated the search for more natural osteoprotective compounds that have fewer side effects than synthetic drugs.

Many commonly consumed foods, herbs, and spices contain a complex array of naturally occurring bioactive molecules, called phytochemicals, which may confer health benefits[11, 12]. Soy foods and soy-derived isoflavones have received considerable attention for their potential role in preventing ovariectomy (OVX) or menopause-induced osteopenia in rats and women, respectively[13–17]. These naturally occurring selective estrogen receptor modulators have beneficial effects on bone that are similar to those of the drug raloxifene[18, 19].

Very recently, attention has been focused on the potential osteoprotective role of other naturally occurring polyphenols. Two lignans, secoisolariciresinol diglycoside from flaxseed and isotaxiresinol from the yew tree (*Taxus yunnanensis*), were found to prevent bone loss in postmenopausal women and in OVX rats, respectively[20, 21]. Arylnaphthalene lignans isolated from the tabu-no-ki tree (*Machilus thunbergii*) increased mouse osteoblast differentiation, as evidenced by increased alkaline phosphatase (ALP) activity, collagen content, and mineralization[22]. Flavonoids, such as rutin, inhibited estrogen deficiency-induced bone loss in OVX rats, by slowing resorption and increasing osteoblastic activity, resulting in increased femoral strength[23].

Numerous studies have examined the antioxidant activities of some plant-derived phenolic compounds that are potent free-radical scavengers[24, 25]. These compounds are beneficial in preventing bone loss because oxidative stress enhances bone resorption by promoting osteoclastic differentiation[26, 27]. Polyphenolic compounds from the Chinese herb du-zhong (*Eucommia ulmoides Oliver*) exhibit osteoprotective activity as well as antioxidative effects[28–30]. Du-zhong extract can inhibit lipid peroxidation and oxidative damage in biomolecules[31–34]. Good correlation was observed between the polyphenolic content and antioxidant activity level of du-zhong extracts[35]. The most abundant polyphenol in du-zhong is chlorogenic acid (CGA), which is also found in coffee beans, potatoes, and other plants. CGA has various biological effects, including antioxidant, antiobesity, antiapoptosis, and antitumor activities[36–38].

As it already have been reported that the Src homology domain 2 (SH2)-containing tyrosine phosphatase SHP-2, is a ubiquitously expressed non-transmembrane tyrosine phosphatase with two SH2 domains, has been implicated that regulates the phosphatidylinositol 3'-kinase (PI3K)/Akt pathway. The ability of SHP-2 to regulate the PI3K/Akt pathway is suggested to result in the positive effect of SHP-2 on cell survival. SHP2 is required for the full activation of the MAPK/ERK pathway and its catalytic activity regulates the PI3K/AKT pathway resulting in the positive effect of SHP2 on cell survival likely participates in anti-apoptotic signaling by suppressing caspase 3-mediated apoptosis [39,40].

In this study, we investigated whether CGA can prevent OVX-induced osteoporosis in rats and whether such effects are due to the promotion of proliferation and osteoblast differentiation of bone mesenchymal stem cells (BMSCs). The major aim of this study was to determine the mechanism by which if CGA can prevent osteoporosis in OVX rats, an established model of postmenopausal osteoporosis[41, 42]. In particular, because Shp2 can regulate the phosphoinositide 3-kinase (PI3K)/Akt pathway and has been implicated in mitogenic and cell differentiation responses[39], we examined whether Shp2 regulates CGA-induced osteoblast differentiation.

## Materials and Methods

### Animals, treatments, and specimen collection

The study protocol was approved by the Institutional Animal Care and Use Committee of the University of NanChang. Sixty 3-month-old virgin Sprague-Dawley specific-pathogen-free female rats (body weight [BW]:180 ± 12.0 g) were housed at 22°C (air condition) under a 12-h light/dark cycle, housed with the multiple standard rodent cages(545×395×200mm) in a cage rack. Ethical approval for the use of animals in this study was granted by the Animal Research Ethics Committee of China. During the experimental period, all rats were allowed free access to distilled water and standard rat chow. We provided the distilled water and standard rat chow every day, monitoring the animals by way of observing the stable baseline and circadian variation in temperature and activity every day. Acclimatized rats underwent either bilateral laparotomy (sham, N = 10) or bilateral OVX (N = 50) after being anesthetized with an intraperitoneal injection with 3% sodium pentobarbital (50mg/kg). After the surgical procedures, monitoring the animals during the recovery period will help confirm surgical recovery, sham rats were fed with the OVX rats to minimize differences in BW between the two surgical groups. Four weeks after surgery, the OVX rats were divided randomly into five treatment groups (N = 10 per group): OVX plus saline vehicle, OVX plus 17α-ethinylestradiol (E2), and OVX plus one of three CGA doses, 9, 27, and 45 mg/kg/d (CGA9, CGA27, and CGA45, respectively). E2 was used as a reference compound to assess the estrogenic effects on bone and to facilitate comparison of the CGA effects on bone with the estrogenic effects. The study diagram on S1 File. Followed the Human Rat Equivalent Dose Conversion Principle[43, 44], the equivalent doses of CGA and E2 used in the experiment were the corresponding clinical prescription dose range for a 60-kg human subject. CGA, vehicle, and E2 were administered orally through a commerce made gastric tube. Treatments started on week 4 post-OVX and continued for 12 weeks.

The BWs of animals were recorded weekly during the experimental period. One day before the animals were euthanized, each rat was housed individually without food for 24 h in a metabolic cage. A urine sample was collected from each rat, and the urine was acidified with 2 ml of 1 Mm hydrochloric acid (HCL). On the following day, sacrifice was performed when the rat was anesthetized with an intraperitoneal injection with 3% sodium pentobarbital (50mg/kg). A blood sample was collected via abdominal aorta puncture, and serum was prepared by centrifugation (2000 rpm for 20 min). Urine and serum samples were stored at -80°C for biochemical analysis. The organs including brain, lung, heart, liver, spleen, kidney, uterus and thymus were obtained from each rat and weighed immediately after sacrifice. Femurs were dissected and stored in normal saline at -20°C for bone analyses.

### Assessment of BMD and bone microarchitecture

According to the previous description[45], two-dimensional total bone mineral content (t-BMC) and total bone mineral density (t-BMD) of each rat’s right femur were measured by dual-energy X-ray absorptiometry (DXA) with the Lunar Prodigy Advance system (GE Healthcare, USA) that was equipped with appropriate software for bone density assessment in small animal’s laboratory. BMD was calculated using the BMC of the measured area and reported as g/cm^3^.

After the measurement of BMD, selected three representative right femurs from each group to evaluate the trabecular micro-architecture of the femoral metaphysis by micro-computed tomography (μCT, Scanco Medical, Zürich, Switzerland). Selection of the representative samples was based on the median value of t-BMD of the respective group[46, 47]. Because the trabecular bone is rich in the distal femur compared to the proximal and middle regions, scan was performed from the proximal growth plate in the distal direction (16 μm/slice) for every selected femur sample. 350 images were obtained from this region of each femur using a 1024 × 1024 matrix, resulting in an isotropic voxel resolution of 22 μm^3^[47].

The volume of interest (VOI) was selected as a cross-sectional area spanning 25–125 slices from the proximal growth plate. This scanning generated 3D images of the micro-architecture that were examined and displayed. Bone morphometric parameters obtained from the μCT, involving the bone volume fraction (BV/TV), trabecular number (Tb.N), trabecular separation(Tb.Sp), trabecular thickness (Tb.Th), connectivity density (Conn.D), and structure model index (SMI), were analyzed for the VOI. The operator conducting μCT analysis was blinded to the treatments associated with the specimens. All examinations were conducted according to the principles and procedures described in the most recent National Research Council publication of the Guide for the Care and Use of Laboratory Animals and refer to the ARRIVE guidelines[48].

### Serum and urine assays

As described previously[49], The S-Ca (serum calcium), S-P (phosphorus), and ALP concentrations were measured with commercial kits (ZhongSheng BeiKong Bio-technology and Science, PRC) by standard colorimetric methods. These concentrations were measured on a Cobas Integra 400 Plus automatic biochemical analyzer (Roche Diagnostics, Switzerland). Urine calcium (U-Ca), phosphorus (U-P), and creatinine (Cr) concentrations were also detected by standard colorimetric methods. Serum osteocalcin (OC) concentration was tested with a rat OC ELISA kit (Biomedical Technologies, Stoughton, MA, USA), with 4% intra- and 7% interassay variabilities followed by the introduction of the manufacturer. Urinary deoxypyridinoline (DPD) concentration was assayed using a rat DPD ELISA kit (Quidel, San Diego, USA), with intra-and inter-assay variabilities of 5.5% and 3.1%, respectively. Urinary excretions of Ca and DPD were expressed as the ratio to Cr concentration (i.e., Ca/Cr, DPD/Cr).

### Isolation of rat BMSCs

Two-month-old Sprague-Dawley rats of both sexes (BW of ~100 g) were purchased from the Jiangxi Traditional Chinese Medicine Hospital Experimental Animal Center and maintained under specific pathogen-free conditions. All animal experiments complied with the animal protocols approved by the Institutional Review Board of the Second Affiliated Hoapital of NanChang University. Both femurs and tibias were dissected. Bone marrow cells were flushed out with phosphate-buffered saline (PBS) in a 5-ml syringe fitted with a gauge needle. Mononucleated cells were isolated by density gradient centrifugation in rat lymphocyte separation medium (Solarbio, Beijing, China) at a concentration of 1.091 g/ml. Isolated cells were seeded in standard low-glucose Dulbecco’s Modified Eagles Medium (DMEM, Hyclone, Shanghai Bioleaf Biotech Co., Ltd., China), supplemented with 15% fetal bovine serum (FBS, Hyclone, Shanghai, China), and then cultured in a humidified incubator with 5% CO_2_ at 37°C. The medium was changed at every 3 d. When the cells were grown to 80% confluency, they were trypsinized and plated onto new dishes as passage 1. All cells used for the experiments were from passage 3.

### Induction of osteoblast differentiation in rat BMSCs

To induce osteoblast differentiation, 1 × 10^4^ rat BMSCs were seeded in 24-well plates containing osteoblast differentiation medium (ODM; low-glucose DMEM supplemented with 10% FBS, 10^−8^ M-dexamethasone,10 mM β-glycerophosphate, and 50 μg/ml ascorbic acid; all from Sigma-Aldrich, Shanghai, China). Cells exposed to ODM without CGA treatment were used as a positive control. Cells exposed to standard medium without differentiation factors were treated with various concentrations of CGA (Sigma-Aldrich), ranging from 0 μM (control) to 10 μM. All experiments were performed in triplicate.

### ALP activity assay

ALP activity was used as an early marker of osteoblast differentiation. After 7 d of treatment, cells grown in ODM and CGA groups or standard medium were harvested, re-suspended in 250 μl of culture medium, and then subjected to ultrasonic cell lysis. After centrifugation, ALP activity in the cell supernatants was quantified with an ALP detection kit (Bio-Rad Laboratories Inc., Hercules, CA). The optical density of each sample was measured on a spectrophotometer at 520 nm. Each value was normalized to the protein concentration.

### Cell proliferation assay (MTT assay)

The viability of cells was measured on the basis of their ability to reduce tetrazolium salts via mitochondrial dehydrogenases. The degree of color development correlates with the number of live cells. Briefly, cells were inoculated into 96-well plates at a concentration of 1 × 10^4^ cells/ml/well and cultured with 200 μl of DMEM medium per well (n = 3 per group). After 24 h of incubation to allow adherence to the well bottoms, cells were treated with various concentrations of CGA (0–100 μM). After incubation for 72 h, 20 μl of MTT(3-(4,5-dimethyl-2-thiazolyl)-2,5-diphenyl-2-H-tetrazolium bromide) solution (5 mg/ml, Beyotime Institute of Biotechnology, Shanghai, China) were added to each well. After 4 h of incubation, the medium in each well was carefully discarded, a 150-μl aliquot of dimethyl sulfoxide (DMSO) was added to each well, and the samples were agitated at a speed of 50 oscillations/min for 10 min. The optical density of each well was measured at 450 nm by a Universal Microplate Reader (BioTek, Winooski, VT). Medium-containing wells without cells were used as blanks. All measurements were performed in triplicate.

To determine whether CGA can promote the proliferation of osteoblast precursors by activating the PI3K/Akt cell signaling pathway, we studied the effect of 10 μM CGA on BMSC growth in phenol red-free medium containing 1% FBS over a 48-h period. Cells were treated with CGA, with or without concurrent exposure to the PI3K/Akt inhibitor LY294002 (20 mM/L). Cell proliferation was analyzed with the MTT assay described above. To determine whether the cell signaling protein, Shp2, can regulate the effect of CGA on osteoblast differentiation, BMSCs in phenol red-free medium containing 1% FBS were transfected with 10 μM of either control or Shp2 interfering RNA (RNAi, 3 μL for 48 h). The control group consisted of cells exposed to neither Shp2 RNAi nor CGA. Cell proliferation was analyzed with the MTT assay at the end of the transfection period.

### Western blots

Cells were washed twice with PBS and lysed in ice-cold RIPA buffer (50 mM Tris, pH 7.4, 150 mM NaCl, 1.0% NP-40, 0.5% sodium deoxycholate, and 0.1% SDS) supplemented with protease inhibitor cocktail (#11836170001) and PhosStop (#4906845001) from Roche (Nutley, NJ). Protein content was quantified with BCA protein assay kits (Pierce, Rockford, IL), according to the manufacturer’s instructions.

For western blot analysis, samples containing 30–50 μg of protein were separated by sodium dodecyl sulfate-polyacrylamide gel electrophoresis and transferred to polyvinylidene difluoride membranes. Using standard blotting techniques, membranes were treated with the following antibodies: phospho-Akt (S473, #4060), Akt (#2967), cyclin D1 (#2926)(Cell Signaling Technology, Beverly, MA); Sh-p2 (C18), β-actin (sc-47778), to quantify the proteins of p-Akt, Akt, cyclin D1 and Sh-p2 for CGA inducing BMSC proliferation via PI3K/Akt/cyclin D1 signaling and CGA regulating osteoblast differentiation of BMSCs via the Shp2/PI3K/Akt pathway, goat anti-mouse IgG-HRP, and goat anti-rabbit IgG-Biotin (Santa Cruz Biotechnology, Dallas, TX). Each primary antibody (1:1000 to 1:5000 dilution) and conjugated secondary antibody (1:3000 dilution) was diluted with Tris-buffered saline with Tween (0.01 M NaCl, 0.025 M Tris-Cl, and 0.5% Tween-20) containing 5% bovine serum albumin. Immunolabelling was visualized by application of the ECL Plus detection system (Bio-Rad, USA).

### Data analysis

All values were expressed as the mean standard deviation (SD). Data analysis was performed with SPSS 13.0, one-way analysis of variance (ANOVA) followed by Student's t-test was used to determine statistically significant differences between groups. Differences with a p-value less than 0.05 were considered statistically significant. All the primer date can refer to the S1 dateset.

## Results

### CGA does not reverse OVX-induced changes in BW or uterine weight

**Fig 1:**
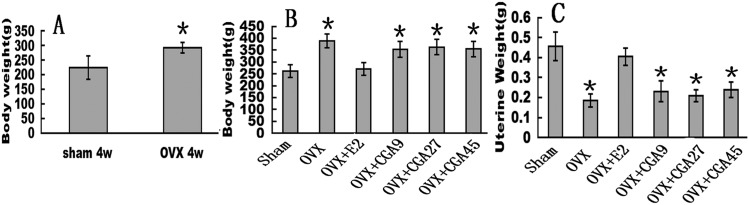
Effects of OVX, E2, and CGA on body and organ weights. OVX rats were treated for a total of 12 wks. Each column represents the mean ± SD of 10 rats. *p < 0.01 vs. sham group. (A) Body weight at week 4 post-OVX or -sham procedure. (B) BW of OVX rats were treated with E2 or CGA for 12 wks. *p < 0.05 vs. sham group. (C) Uterine weight of OVX rats given E2 or CGA. *p < 0.01 vs. sham group.

All groups of rats had similar initial mean BWs. Despite pair feeding, the mean BW of the OVX group was higher than that of the sham group at all time points from week 4 postsurgery onward (Fig 1A). E2 prevented the increase in BW associated with OVX-induced hormone deficiency. The mean BW of the OVX group recovered to that of the sham group by 12 weeks post-treatment with E2 (Sham:262.00±26.68; OVX: 389.696±29.074; OVX+E2:270.488±27.895; OVX+CGA9:353.144±33.287; OVX+CGA27:362.406±32.591; OVX+CGA45:354.367±31.774) (*p* < 0.05). None of the CGA doses had affected the OVX effect on BW (Fig 1B). Mean uterine weight was decreased after OVX compared to that in sham controls (Sham:0.456±0.072; OVX: 0.184±0.032)(*p* < 0.01), indicating successful OVX. E2 increased the mean uterine weight compared to that of the OVX group (OVX+E2:0.404±0.042)(*p* < 0.01). CGA did not elicit any uterotrophic effects (OVX+CGA9:0.23±0.051; OVX+CGA27:0.208±0.03; OVX+CGA45:0.239±0.037) (Fig 1C). Mean weights of the heart, liver, spleen, lung, kidney, and brain were not significantly different among the sham and treatment groups (the figures has not been displayed).

### CGA promotes bone formation

**Fig 2:**
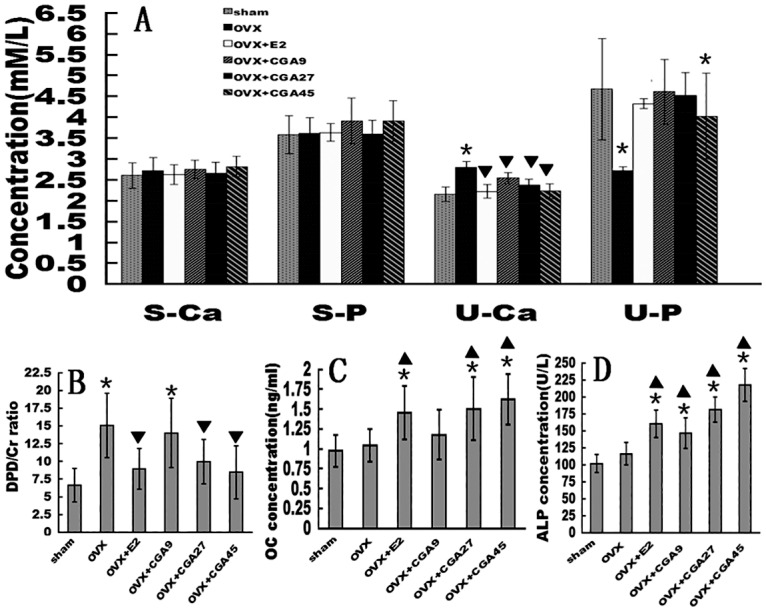
Effects of E2 and CGA on biochemical markers of bone remodeling in OVX rats. Rats were treated for a total of 12 wks. Each column represents the mean ± SD of 10 rats. *p < 0.01 vs. sham group. ▼decrease vs. OVX group (*p* < 0.01) ▲ increase vs. OVX group (*p* < 0.01). (A)S-Ca, S-P, U-Ca, and U-P of OVX rats in response to E2 and CGA. (B) Urinary DPD/Cr ratio of OVX rats in response to E2 and CGA. (C) Serum OC of OVX rats in response to E2 and CGA. (D) Serum ALP activity of OVX rats in response to E2 and CGA.

There were no significant differences in mean S-Ca or S-P among the groups (Fig 2A). Mean U-Ca (Sham:2.149±0.169; OVX: 2.801±0.14; OVX+E2:2.222±0.165; OVX+CGA9:2.538±0.129; OVX+CGA27:2.379±0.142; OVX+CGA45:2.228±0.183) was higher and mean U-P (Sham:4.673±1.221; OVX: 2.721±0.089; OVX+E2:4.325±0.121; OVX+CGA9:4.609±0.772; OVX+CGA27:4.518±0.553; OVX+CGA45:4.027±1.024) was lower in the OVX group than in the sham group (*p* < 0.01 for both analyses). All three CGA doses prevented the OVX-induced increase in U-Ca in an apparently dose-dependent manner (all *p* < 0.01). Low and moderate CGA doses prevented the OVX-induced decrease in mean U-P; the highest CGA dose resulted in a significant U-P difference compared to E2 group. E2 treatment had an effect similar to that of CGA on OVX-induced U-Ca increase and OVX-induced U-P decrease (all *p* < 0.01 vs. OVX).

At 12 weeks post-OVX, the mean urinary DPD/Cr ratio, a bone resorption marker[50], was higher in all five OVX subgroups than that of the sham group, but the mean urinary DPD/Cr ratios of the E2, CGA27, and CGA45 groups were significantly decreased (Sham:6.646±2.347; OVX: 15.071±4.542; OVX+E2:8.938±2.898; OVX+CGA9:14.005±4.9; OVX+CGA27:9.95±3.118; OVX+CGA45:8.454±3.749) (Fig 2B). The CGA effect on the DPD/Cr ratio appeared to be dose-dependent. Levels of the bone formation markers, OC[51] and ALP activity[52], increased with CGA treatment. The mean OC level was increased in the CGA27 and CGA45 groups compared to levels in the sham and OVX groups (OVX:1.044±0.203; OVX+E2:1.452±0.335; CGA9:1.177±0.311; CGA27:1.505±0.399; CGA45:1.619±0.317) (all *p* < 0.01, Fig 2C). At all doses, CGA treatment increased serum ALP activity (OVX: 116.137±16.348; OVX+E2:157.975±20.1; CGA9:146.412±22.82; CGA27:180.977±18.358; CGA45:217.665±24.114) (all *p* < 0.01 vs. OVX) in a dose-dependent manner (Fig 2D). E2 treatment had a significant effect similar to that of CGA27, CGA45 on OC and ALP activity levels (Fig 2C and 2D).

### CGA increases femoral BMD

**Fig 3:**
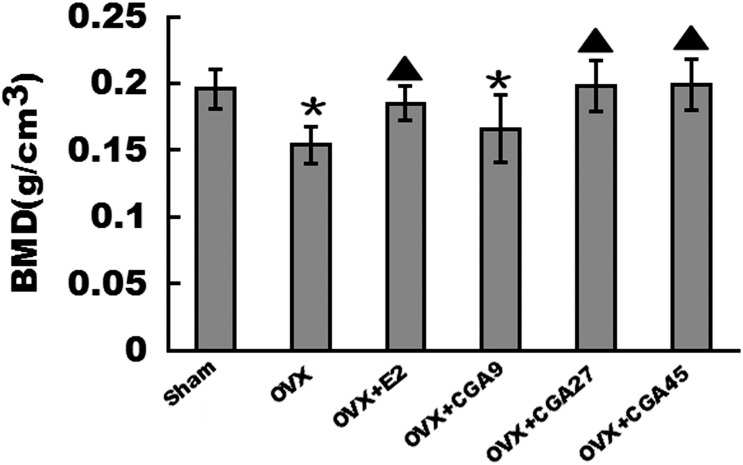
Effect of E2 and CGA on BMD in OVX rats. Each column represents the mean ± SD of 10 rats. *p < 0.01 vs. sham group. ▲ increase vs. OVX group (*p* < 0.01).

The mean BMD of the OVX group was lower than that of the sham group (*p* < 0.01, Fig 3). Mean right femur BMD values were increased in the E2, CGA27, and CGA45 groups compared to the OVX group (Sham: 0.195±0.014; OVX:0.154±0.015; OVX+E2:0.185±0.013; CGA27:0.198±0.018; CGA45:0.199±0.019) (*p* < 0.01). There were no significant differences in the mean right femur BMD values among the E2, CGA27, and CGA45 groups.

### CGA improves bone microarchitecture

**Fig 4:**
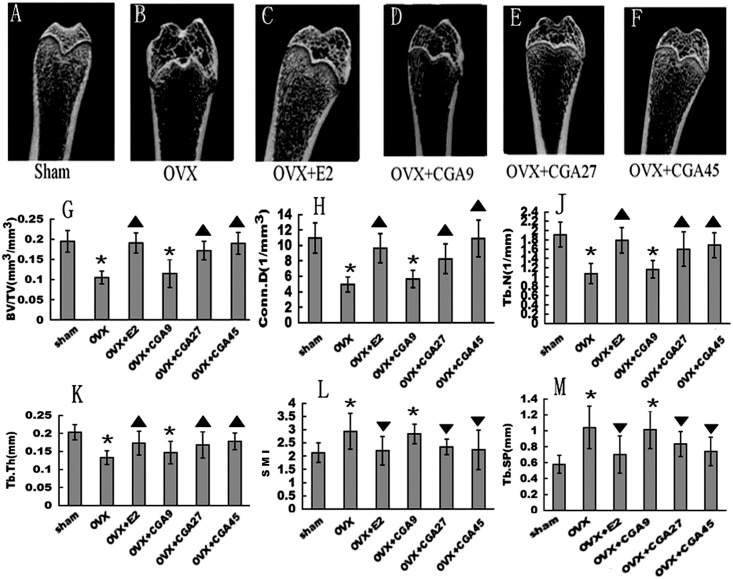
Positive effects of E2 and CGA on parameters of bone microarchitecture in OVX rats. *p < 0.01 vs. sham group. (A–F) μCT images showing representative trabecular bone microarchitecture of the femoral metaphysis for each treatment group. (G–K) Effects of E2 and CGA on BV/TV, Conn.D, Tb.N, and Tb.Th in OVX rats. ▲ increase vs. OVX group (*p* < 0.05). (L and M) Effects of E2 and CGA on SMI and Tb.Sp in OVX rats. ▼decrease vs. OVX group (*p* < 0.05).

Three-dimensional images of femoral metaphyses generated by μCT showed differences in trabecular micro-architecture among the various treatment groups (Fig 4A–4F). Analysis of data from the representative samples indicated that OVX decreased trabecular BV/TV(Sham:0.194±0.026; OVX: 0.105±0.016; OVX+E2:0.191±0.025; OVX+CGA9:0.114±0.035; OVX+CGA27:0.171±0.023; OVX+CGA45:0.190±0.027), Conn.D (Sham:10.952±1.945; OVX: 4.973±0.992; OVX+E2:9.635±1.856; OVX+CGA9:1.119±0.354; OVX+CGA27:8.257±1.915; OVX+CGA45:10.893±2.361), Tb.N (Sham:1.914±0.272; OVX: 1.072±0.217; OVX+E2:1.784±0.274; OVX+CGA9:1.165±0.184; OVX+CGA27:1.599±0.367; OVX+CGA45:1.686±0.268), and Tb.Th (Sham:0.202±0.020; OVX: 0.132±0.019; OVX+E2:0.172±0.033; OVX+CGA9:0.146±0.030; OVX+CGA27:0.168±0.036; OVX+CGA45:0.177±0.023) (all *p* < 0.01), compared to values obtained for the sham group (Fig 4G–4K). By contrast, SMI (Sham:2.129±0.369; OVX: 2.933±0.679; OVX+E2:2.203±0.535; OVX+CGA9:2.836±0.374; OVX+CGA27:2.345±0.297; OVX+CGA45:2.236±0.741) and Tb.Sp (Sham:0.577±0.112; OVX: 1.041±0.266; OVX+E2:0.702±0.234; OVX+CGA9:1.012±0.235; OVX+CGA27:0.832±0.158; OVX+CGA45:0.740±0.178)in the proximal femur were increased (both *p* < 0.01) in response to OVX, compared to values obtained for the sham group (Fig 4L and 4M). All of these OVX effects were reversed in the E2, CGA27, and CGA45 groups (all *p* < 0.05 vs. OVX).

### CGA induces osteoblast differentiation

**Fig 5:**
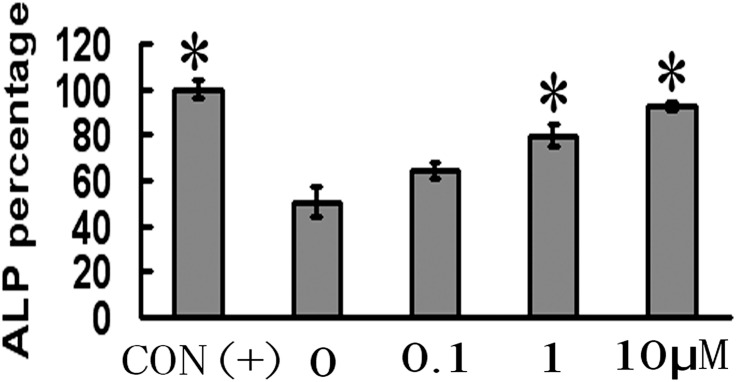
Effect of CGA on osteoblast differentiation of rat BMSCs. Each column represents the mean ± SD of optical density values. CON (+) cells were exposed to ODM without CGA. 0 μM group represents cells exposed to neither ODM nor CGA. *p < 0.01 vs.

ALP activity in BMSCs showed a positive dose-dependent response to increasing CGA concentrations from 0.1 to 10 μM (CON:1.189±0.040; CGA0: 0.596±0.065; CGA0.1:0.765±0.033; CGA1:0.946±0.048; CGA10:1.102±0.021)(Fig 5). ALP activity levels in BMSCs following 1 μM or 10 μM CGA treatment were higher (*p* < 0.01) than those in BMSCs exposed to neither ODM nor CGA (0 μM, control). There were no significant differences in ALP activity between BMSCs treated with CGA (1.0 μM or 10 μM) and those exposed to ODM (positive control).

### CGA induces BMSC proliferation via PI3K/Akt/cyclin D1 signaling

**Fig 6:**
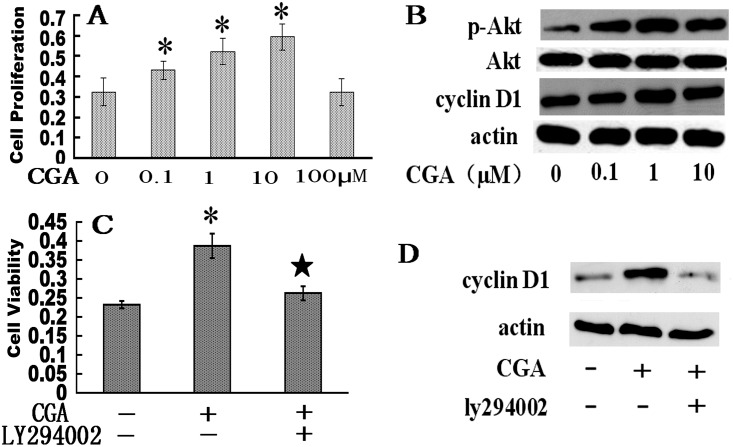
Effect of CGA on proliferation of rat BMSCs. (A) MTT assay of cell viability. Each column represents the mean ± SD of optical density values.*p < 0.05 vs. 0 μM group. (B) Western blots of p-Akt, Akt, and cyclin D1expression in cells exposed to CGA. β-Actin was used as the loading control. (C) Viability in cells exposed (+) to 10 μM CGA and 2 μM LY294002 (PI3K/Akt inhibitor). Each column represents the mean ± SD of the optical density values. *p < 0.05 vs. control (–/–) group. ★*p* < 0.05 vs. CGA+/ LY294002+ group. (D) Western blot of cyclin D1 expression in cells exposed (+) to 10 μM CGA and 2 μM LY294002. β-Actin was used as the loading control.

Proliferation of BMSCs exhibited a positive dose-dependent response to increasing CGA concentrations (CON:0.323±0.067; CGA0.1:0.430±0.043; CGA1:0.522±0.064; CGA10:0.593±0.064; CGA100:0.323±0.065) (range, 0.1–10 μM). Proliferation of BMSCs treated with 1 μM or 10 μM CGA was increased compared to that of untreated control BMSCs (*p* < 0.05; Fig 6A). Proliferation of BMSCs was inhibited with 100 μM CGA, indicating that this dose may have had a cytotoxic effect. Akt phosphorylation and cyclin D1 expression showed a positive dose-dependent response to CGA (range, 0.1–1 μM; Fig 6B) and that also be enhanced to CGA 10 μM. CGA’s enhancing effects on phosphorylated Akt (p-Akt) and cyclin D1 were diminished by concurrent exposure to the specific PI3K/Akt inhibitor LY294002 (Fig 6C and 6D).

### CGA regulates osteoblast differentiation of BMSCs via the Shp2/PI3K/Akt pathway

**Fig 7:**
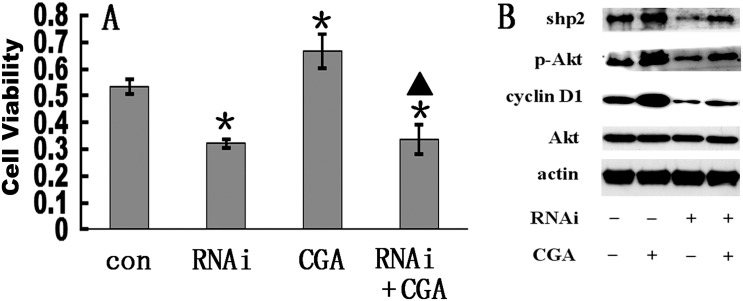
Effect of Shp2/PI3K/Akt/cyclin D1 pathway inhibition on CGA-induced upregulation of osteoblast differentiation in rat BMSCs. (A) Viability in cells exposed to 10 μM Shp2 RNAi and 10 μM CGA. Each column represents the mean ± SD of the optical density values. *p < 0.01 vs. control (con) group. ▲ p < 0.01 vs. RNAi group. (B) Western blots of Shp2, p-Akt, cyclin D1, and Akt expression in cells exposed (+) to 10 μM CGA and RNAi. β-actin was used as the loading control.

Transfection of BMSCs with10 μM Shp2 RNAi reduced cell proliferation compared to that of untreated control cells (CON:0.323±0.009; CGA10:0.387±0.032; CGA10+LY294002:0.262±0.019) (*p* < 0.01), and also reduced the enhancing effect of CGA on cell proliferation (Fig 7A). Expression of Shp2 was enhanced by CGA, but it was suppressed by Shp2 RNAi in cells exposed, as well as cells not exposed, to CGA compared to expression levels in mock-transfected BMSCs (Fig 7B).

## Discussion

In this study, the results suggeste that CGA improved osteoporosis(BMD) and promoted bone formation markers(OC and ALP) in OVX rats. In agreement with previous observations[47], we found that OVX-induced hormone deficiency significantly increased BW, and, as expected, this excess BW gain was completely prevented by E2 administration. Although the exact mechanism by which OVX induces weight gain is not clear, estrogen may be directly involved in energy metabolism through binding to ERs within the abdominal, subcutaneous, and brown fat pads[53, 54]. CGA at all dose levels tested did not prevent the increase in BW induced by OVX in rats. These results suggest that CGA at the dosages tested does not behave like E2 in the regulation of BW and uterine tissue growth in OVX rats. In this study, there are two rats died unexpectedly and without intervention within two days after laparotomy, we add another two rats with laparotomy, it may be we have not master the laparotomy technology at the initiation. We found that there is no side effects occured in all these rats such as breast cancer,cardiovascular disease, VTE and upper gastrointestinal symptoms. It suggested that the CGA may be safe for treatment of osteoporosis.

We also evaluated the effects of CGA on specific parameters of bone mineralization, formation, and micro-architecture in mature female rats to confirm and further characterize CGA’s *in vivo* osteogenic effects. As expected, OVX resulted in a decreased femur t-BMD after 16 weeks. This loss of bone mass was accompanied by a significant increase in bone remodeling, as evidenced by an enhanced level of the bone turnover marker DPD. Treatment with CGA dose-dependently prevented the OVX-induced decrease in t-BMD, which was reflected by increases in serum OC and ALP levels, and a decreased urine DPD/Cr ratio. Decreasing the fecal and urinary excretion of calcium and increasing the calcium absorption efficiency might help to prevent reduction of BMD[55, 56]. In our study, CGA dose-dependently prevented the OVX-induced increase in U-Ca excretion, and CGA at low and moderate doses (9 and 27 mg/kg/d) attenuated OVX-induced decreases in U-P excretion(*p* < 0.05), but the effect of attenuation on OVX-induced decrease in U-P excretion at CGA 45mg/kg/d dose is not so significant(sham:4.673±1.221; OVX:2.721±0.089;OVX+CGA45:2.827±0.145) (*p*> 0.05) as low and moderate doses(we have not detected the reasons). These CGA effects mirrored those observed with E2 and are consistent with the maintenance of bone mass by inhibiting bone remodeling.

Although inhibition of bone remodeling would be considered beneficial generally, the biomechanical competence of bone might be decreased if bone remodeling were to be inhibited for an excessive period of time. For instance, treatment with pamidronate (14 mg/kg/d for 25 d) has been shown to decrease intrinsic diaphyseal bone strength in rats[57]. Because BMD is considered only a surrogate measure of bone strength[58], micro-architecture determinants are necessary to evaluate the true impact of a treatment on trabecular bone quality because trabecular bone is more readily lost in the OVX rodent model[59]. Preservation of the trabecular micro-architecture contributes to bone strength and may reduce fracture risk independently of BMD and BMC[60, 61]. We found that CGA27 and CGA45 significantly improved all parameters of trabecular micro-architecture in the femurs of OVX rats.

The SMI is used to distinguish between rods and plates in trabecular bone. Khajuria reported that SMI grades of 0 and 3 represent bone that consists purely of plate- or rod-like structures, respectively[59]. Analyzed from our results indicate that is a moderate but significant transition of trabecular structures from rods to a mixed plates-and-rods form in OVX rats treated with CGA, especially at the high dose. None of the CGA doses or E2 was able to restore trabecular bone completely, although CGA had positive effects on trabecular micro-architectural properties. These findings are in accord with those of previous researches [60, 62] that was not observed complete restoration of trabecular structure after its deterioration had occurred. This finding emphasizes the need for prevention of trabecular bone loss.

To prove the mechanism by which CGA prevents OVX-induced bone loss in mature rats, the effect of CGA on osteogenesis was investigated *in vitro*. We demonstrated that CGA can promote BMSC proliferation and induce osteoblast differentiation. After inhibiting several key cell signaling pathways, such as PI3K/Akt and MAPK (Erk1/2), we found that the major signaling pathway involved in BMSC proliferation PI3K/Akt. Inhibition of only this pathway reversed the stimulatory effect of CGA on BMSC proliferation. We further demonstrated that CGA exerts its proliferative effect on BMSCs by activating cyclin D1, which acts downstream of PI3K/Akt. Upregulation of cell proliferation by CGA was blocked by Akt or Shp2 inhibition. Shp2, a major cytoplasmic tyrosine phosphatase regulates the PI3K/Akt pathway. Our findings indicate that the Shp2/PI3K/Akt pathway is involved in the upregulation of osteoblast differentiation by CGA. Our finding that Shp2 RNAi inhibited the CGA-induced osteoblast differentiation of BMSCs suggests the Shp2 may be a key regulator of CGA’s influence on osteoblast differentiation.

In addition to reducing oxidative stress, CGA might inhibit bone resorption in a similar manner to phytoestrogens, that is, by competing with estrogen for estrogen receptors (ERs)[63]. Rat, mouse, and human ERs exist as two subtypes, α and β. The ERβ subtype is more abundant than the ERα subtype in bone tissue. ERα is mainly distributed in reproductive cells, especially distributed in those of the breast and uterus. Our finding that CGA can increase BMD and improve bone micro-architecture without increasing uterine weight, similar to E2, suggests that CGA might have a higher affinity for ERβ than for ERα. If so, CGA might be clinically useful for preventing bone loss without stimulating unwanted proliferation of the uterine tissues.

Dang et al.[64] reported that estrogen plays an important role in stimulating the differentiation of progenitor cells through the osteoblast lineage. Another study revealed ERβ-like immunoreactivity in the nuclei of human and murine osteoblasts and osteocytes and in the cytoplasm of osteoclasts and chondrocytes[65]. ERβ mRNA is present in rat osteoblasts, predominantly those covering the metaphyseal bone trabecular surface[66]. Our study showed that CGA found in du-zhong has a direct stimulatory effect on the proliferation and differentiation of cultured rat osteoblast precursors in vitro. Thus, it is plausible that CGA can stimulate osteoblastic activity and inhibit osteoclastic resorption via binding to ERβ. But the binding relationship should continue to be researched.

## Conclusion

Daily oral administration of CGA over a 12-week period can prevent estrogen deficiency-induced bone loss and deterioration of trabecular micro-architecture in the adult OVX rat, thereby maintaining the biomechanical competence of bone. CGA exerts these positive effects on bone remodeling by stimulating proliferation of osteoblast precursors via the PI3K/Akt/cyclin D1 pathway and by upregulating osteoblast differentiation via the Shp2/PI3K/Akt/cyclin D1 pathway. Moreover, CGA does not behave as E2 on the uterus, indicating that CGA might not have the same side effects as HRT, such as breast cancer, and orther risk factors for cardiovascular disease and VTE. CGA was detected in high amount in *Eucommia ulmoides*(*duzhong)* which is a functional food and drink in China. As the major component of an herb used in traditional Chinese medicine, CGA has the potential to be a safe and effective alternative medical approach to preventing or treating postmenopausal osteoporosis. Clinical trials are warranted to determine the effectiveness of CGA in improving bone remodeling in postmenopausal patients.

## Supporting Information

S1 File Study diagram. This diagram describe the design of our performation of the study.
(DOC)

S1 Dataset Include all the primer date. (7Z)
