# Supplementary material for: Chlorogenic Acid Prevents Osteoporosis by Shp2/PI3K/Akt Pathway in Ovariectomized Rats
Source: PLoS One. 2016 Dec 29;11(12):e0166751. doi: 10.1371/journal.pone.0166751 (PMC5199056; doi:10.1371/journal.pone.0166751)
Supplement: S1 File — This diagram describe the design of our performation of the study. (DOC) [file pone.0166751.s001.doc]

- Study diagram.

*In vivo*

*In vitro*

Sham (10)

OVX(50)

Osteoblast

differentiation

proliferation

4W

signaling pathway

Sham (10)

OVX(10)

OVX+E2(10)

OVX+CGA9(10)

OVX+CGA27(10)

OVX+CGA45(10)

CGA

0 μM

0.1μM

1μM

10μM

100μM

Con CGA

ODM 0 μM

0.1μM

1μM

10μM

CGA

0 μM

10μM

10μM+

Ly294002

12W

U-Ca S-Ca body W

U-P S-P organ W

U-Cr S-OC BMD

U-DPD S-ALP uCT

ALP

MTT

SHP2 RNAi

or CGA

p-Akt

Akt

cyclin D1

Sh-p2

S1 File: Study diagram: this diagram describe the design of our performation of the study
